# Use of biological mesh versus standard wound care in infected incisional ventral hernias, the SIMBIOSE study: a study protocol for a randomized multicenter controlled trial

**DOI:** 10.1186/1468-6708-14-131

**Published:** 2013-05-07

**Authors:** Christophe Mariette, Nicolas Briez, Fanette Denies, Benoît Dervaux, Alain Duhamel, Marie Guilbert, Emilie Bruyère, William B Robb, Guillaume Piessen

**Affiliations:** 1 Department of Digestive and Oncological Surgery, University Hospital of Lille, Place de Verdun, Lille Cedex, 59037, France; 2 University Lille Nord de France, Place de Verdun, Lille Cedex, 59045, France; 3 Inserm UMR 837, Team 5 Mucins, Epithelial Differentiation and Carcinogenesis, JPARC, Rue Polonovski, Lille Cedex, 59045, France; 4 Hospital Pharmacy, University Hospital of Lille, Place de Verdun, Lille Cedex, 59037, France; 5 Department of Biostatistics and Public Health, University Hospital of Lille, Place de Verdun, Lille Cedex, 59037, France; 6 Department of Digestive and Oncological Surgery, University Hospital Claude Huriez, Regional University Hospital Center, Place de Verdun, Lille Cedex, 59037, France

**Keywords:** Incisional ventral hernia, Infection, Biological mesh, Randomized trial

## Abstract

**Background:**

In infected incisional ventral hernias (IVHs), the use of a synthetic non-absorbable mesh is not recommended and biological meshes hold promise. However, the level of evidence for their safety and efficacy remains low.

**Methods:**

The SIMBIOSE trial is a multicenter, phase III, randomized, controlled trial comparing the use of a biological mesh versus traditional wound care in patients with an IVH. The primary end point is 6-month infectious and/or wound morbidity. Secondary end points are wound infection and recurrent hernia rates, post-operative pain, quality of life, time to heal, reoperation need, impact of the cross-linked mesh structure, and a medico-economic evaluation. One hundred patients need to be included.

**Results:**

The main results expected with biological mesh use are a significant decrease of post-operative morbidity, hernia recurrence, time to heal, and costs with an improved quality of life.

**Conclusions:**

For the first time, the impact of biological meshes in the treatment of IVHs will be evaluated in an academic, randomized, phase III trial to provide scientific evidence ( NCT01594450).

**Trial registration:**

ClinicalTrial.gov, NCT01594450

## Background

Incisional ventral hernias (IVHs) occur in around 10% to 20% of patients after abdominal surgery [1]. Their repair with a synthetic non-absorbable mesh to reinforce the abdominal wall leads to a significant decrease of nearly 50% in the hernia recurrence rate [2-4].

However, synthetic non-absorbable meshes carry a risk of infection. A grading system has been published by the Ventral Hernia Working Group (VHWG), which can be used to assess the infection risk [5] and to choose the ideal surgical procedure for the repair. The highest risk (Grade 4) occurs in ventral hernias that are characterized by active infection and septic dehiscence, especially if associated with an infected mesh, abscess or enterocutaneous fistula. In those patients, synthetic non-absorbable meshes are not recommended because of the high rates of mesh infection and the subsequent need for mesh removal and the consequent high rates of recurrence [6].

This group of patients is difficult to manage because of the opposing objectives of treating the active infection and repairing the incisional hernia. Solely treating the active infection without a mesh puts the patient at risk of hernia recurrence, and the delayed placement of such a mesh does not protect against recurrent infection [5]. As a result, the primary treatment of a ventral hernia in an infected field frequently requires the use of a synthetic absorbable mesh, with high recurrence rates due to rapid mesh reabsorption and a continued, albeit reduced, risk of mesh colonization and infection [6]. Patients with an infected ventral hernia, whatever primary treatment is chosen, are subjected to repeated operations, prolonged treatment with significant costs, and an impaired quality of life [7].

Biological meshes have been developed for these infrequent and difficult situations. They appear to be ideal in treating the incisional hernia and decreasing the risk of a colonizing infection. The non-human collagen matrix can support tissue regeneration through neovascularization and cell repopulation in a clinically acceptable timeframe and is resistant to infection [5,8]. Biological meshes are recommended by the VHWG in infected fields, but the evidence for their systematic use remains low, due to the reported small patient series, a lack of long-term follow-up, the absence of substantive comparative studies, a high frequency of industrial study sponsors and there are no published, randomized controlled trials [5]. Despite their high cost and the lack of evidence, the use of biological meshes in the treatment of infected incisional hernias has increased greatly in recent years. This has been in part due to the lack of a satisfactory alternative treatment for these difficult cases. Moreover, recent publications have given some of the limitations of bioprosthetic meshes, which have high rates of recurrent hernias [9,10]. There is consequently an urgent need for a robust comparison of biological meshes with standard wound care for patients with an active infection of a incisional ventral hernia evaluating both patient and economic end points.

Consequently, we have designed an academic, large, multicenter, phase III, prospective, randomized, controlled trial comparing the use of a biological mesh versus standard wound care without a biological mesh in patients with an infected incisional ventral hernia, with an associated medico-economic evaluation, the SIMBIOSE trial.

## Methods/design

### Basic protocol overview

The SIMBIOSE trial is a large, multicenter, phase III, prospective, randomized, controlled, single-blinded trial comparing the use of a biological mesh versus traditional wound care without a biological mesh in patients with an infected incisional ventral hernia.

All patients will undergo an initial surgical procedure to treat the wound or intra-abdominal infection, and, either during the same procedure (single stage) or during a second later procedure within 1 month (double stage), repair the incisional ventral hernia.

Randomization is performed during the pre-operative consultation, after confirming the absence of contraindications to inclusion. Arm A patients undergo the implantation of a biological mesh (after debridement and treatment of the infection, either at the same time or within 1 month of randomization). Arm B patients undergo traditional wound care (debridement and treatment of infection), without the implantation of a biological mesh. For arm B, common wound care is used, following the normal practice of each surgeon. A biological mesh will not be placed within 6 months of randomization in this arm. Beyond this period, in the absence of wound healing, the treating surgeon may use a biological mesh as required.

Patients are blinded to the randomization arm during the first 6 months, which is until the evaluation of the primary end point.

To test the hypothesis that biological meshes will reduce abdominal complications, the two groups will be compared regarding their post-operative course and long-term follow-up including medical and economic end points.

This study is planned to last for 5 years, with a 2-year inclusion period and a 3-year follow-up period. The results for the primary end point will be available 6 months after the end of the inclusion period (2 years).

### Objectives

The primary objective is to compare the effect of two therapeutic strategies in the treatment of infected ventral hernias on 6-month infectious and/or wound post-operative morbidity. The two strategies for comparison are the use of a biological mesh versus standard wound care without a biological mesh.

Secondary objectives are the assessment of (i) wound infection rates at 45 days, 3 months and 1 year, (ii) recurrent hernia rates at 1, 2 and 3 years, (iii) post-operative pain, (iv) quality of life, (v) time to heal, (vi) need for wound reoperation due to infection or hernia recurrence, (vii) impact of the cross-linked mesh structure on (a) the primary objective, (b) the 1-year infection rate and (c) 1- and 3-year recurrence rates, (viii) medico-economic evaluation taking into account the direct costs of infected ventral hernia treatment.

### Inclusion criteria

The study includes patients with an active infection of an incisional abdominal wall hernia.

The inclusion criteria are that patients: (i) have a wound infection related to a synthetic non-absorbable mesh of at least 15 days’ duration, (ii) have an incisional abdominal hernia with an abscess or fistula, without the presence of a synthetic non-absorbable mesh, (iii) have an incisional abdominal hernia smaller than 20 cm for the two largest diameters, (iv) have an incisional abdominal hernia requiring surgical repair, (v) have an incisional abdominal hernia amenable to repair with a single biological mesh, (vi) are aged over 18, (vii) have signed written informed consent, and (viii) are entitled to care within the French national public health-care system.

### Exclusion criteria

The general exclusion criteria are the common contraindications for surgery, depending on patient, disease or operative technique. Specifically, exclusion criteria are that patients: (i) do not have an infected incisional abdominal hernia, (ii) have a history of biological mesh placement, (iii) have an incisional abdominal hernia in a contaminated but uninfected field (stoma presence, violation of gastrointestinal tract, or defined as grade 3 by the VHWG), (iv) have an incisional abdominal hernia larger than 20 × 20 cm, (v) have a body mass index ≥ 40 kg/m^2^, (vi) score 4 or 5 on the American Society of Anesthesiologists scale, (vii) have immunosuppression, including ongoing steroid and cytotoxic therapy, (viii) have a chronic disease such as cirrhosis, renal insufficiency with renal dialysis, malignant disease or a known collagen disorder, (ix) have a life expectancy less than 36 months, (x) are allergic to one of the biological mesh components, (xi) are pregnant or breastfeeding, (xii) have refused to participate, (xiii) are non-compliant or (xiv) suffer from legal incapacity.

### Criteria for evaluation of trial objectives

The primary objective will be assessed by the proportion of patients experiencing at least one morbid wound event within 6 months of surgery. Such morbidity includes infectious morbidity (superficial, deep or systemic) and/or wound morbidity (wound dehiscence or recurrence of incisional hernia).

Superficial complications include hematoma or seroma formation, wound abscesses or other problems with wound healing. Wound complications include wound dehiscence, recurrent hernias, mesh infection or associated hematoma. The development of an enterocutaneous fistula, intra-abdominal abscess, peritonitis or intestinal occlusion will be recorded as manifestations of intra-abdominal complications. Other complications of a general nature, including respiratory, cardiac, thromboembolic, septic and immune- or allergy-mediated morbidity, will also be recorded.

A standardized definition of all complications has been given in detail in the protocol to allow uniform reporting.

To evaluate the secondary objectives the following criteria will be evaluated: (i) the proportion of patients with a wound infection at 45 days, 3 months and 1 year after the initial operation; (ii) the proportion of patients with a recurrent hernia on clinical examination 1, 2 and 3 years postoperatively, or on computed tomography (CT) examination 3 years postoperatively; (iii) the proportion of patients suffering post-operative pain at 45 days, 3 and 6 months, and 1, 2 and 3 years as assessed by an analog visual scale and analgesic consumption; (iv) patients’ quality of life using EuroQol 5D, SF-12 and the Carolinas comfort scale (CCS) at 45 days, 3 and 6 months, and 1, 2 and 3 years postoperatively; (v) time to heal, measured between patient randomization and complete wound healing; (vi) the proportion of patients requiring a surgical procedure for a wound event (infection or hernia recurrence) 6 months after randomization, and the number of such surgical procedures; (vii) comparison of cross-linked and non-cross-linked meshes; (viii) medico-economic evaluation at 6 months; (ix) cumulative length of hospital of stay; (x) number of medical consultations; (xi) number of day-case admissions and (xii) related medications taking into account number of prescriptions and amount of drug prescribed.

### Randomization

Patients will be randomized at inclusion during the pre-operative medical consultation after verification of suitability for inclusion. The randomization will be performed by a centralized randomization procedure. Patients will not be informed of the randomization arm during the first 6 months, which is until the time for the primary end-point evaluation.

In arm A, patients will have their hernia repaired with the use of a biological mesh (after debridement and treatment of the infection) at the same time as the primary operation, or within 1 month of randomization.

In arm B, patients will be treated with traditional wound care (debridement and treatment of infection), without placement of a biological mesh. For arm B, the wound care used will follow the normal practice of the treating surgeon. This will include any routine treatment apart from the placement of a biological mesh within 6 months of randomization.

In cases of treatment failure at 6 months, patients in arm B may undergo any treatment which the treating surgeon views suitable, including hernia repair with a biological mesh.

### Pre-operative work

There is a physical examination to assess the location and size of the abdominal wall hernia, whether a synthetic, non-absorbable mesh is already in place, the type of mesh infection (superficial, deep or digestive fistula), failure of healing, pain assessment with an analogical visual scale and patient temperature. As a standard pre-operative biological investigation a wound swab is taken for culturing and to assess sensitivity. There is a pre-operative CT scan to assess: (i) the largest diameter of the hernia, (ii) whether there is a superficial or a deep abscess, (iii) whether there is a digestive fistula and (iv) the intra-abdominal pathology responsible for the wound infection. There is a pre-operative anesthetic consultation.

### Treatment methods

In both arms, surgical procedures should be performed within 30 days of randomization. Irrespective of randomization, the surgery may be performed in one or two steps at the surgeon’s discretion.

For both arms, after excision of the infected wound (as necessary) and intra-abdominal adhesiolysis, an extensive debridement of all infected structures, such as mesh, soft tissues, abscess or fistula, will be performed, aiming to reduce the bacterial load as much as possible. After bacteriological sampling, if a synthetic, non-absorbable mesh is present it will be completely excised, which treats the underlying cause of infection. The abdominal cavity will be lavaged with normal saline.

#### Arm A

A measurement of the two largest diameters of the incisional ventral hernia will be performed, in order to choose the most appropriate size of biological mesh. Intra-operatively the surgeon will decide whether to perform a single-stage procedure, with immediate implantation of the biological mesh, or a two-stage procedure, with a delayed implantation of the biological mesh within the following 30 days.

The biological mesh will be placed in an underlay position, that is, either intra-abdominal or pre-peritoneal retro-muscular, under the appropriate tension, to help prevent the development of laxity. The surgeon is free to choose the biological mesh from among the five biological meshes with a CE mark presently available in France: Collamend FM® (Davol), Strattice® (Lifecell), Tutomesh® (Tutogen), Surgisis® (Cook) and Permacol® (Covidien).

Regardless of placement, the mesh must overlap the intact fascia by at least 3 cm to 5 cm. The mesh fixation will be performed using a monofilament, non-absorbable, polypropylene suture. Where possible, a tension-free closure of the fascia will be performed. Only if fascia closure is impossible, will a bridging repair be used, that is, without a fascia overlay.

Drainage and skin closure will be performed according to the surgeon’s choice.

#### Arm B

Except for the placement of a biological mesh all of the operating surgeon’s usual treatment options will be possible, including long-term wound care, fascial closure with a synthetic absorbable mesh, direct fascial closure without a mesh, negative pressure therapy or autologous skin grafting.

Drainage and skin closure will be performed according to the practices of the operating surgeon.

#### Post-operative period

All patients will benefit from the usual standards of post-operative care. Additional surgical procedures will be performed as required. Other treatments such as negative pressure wound therapy or autologous skin grafting may be used, regardless of the randomization arm.

In arm B, for ethical considerations, after the evaluation of the primary end point at 6 months, in the absence of wound healing and/or persistence of infection, the use of a biological mesh will be possible – if judged necessary by the treating surgeon.

### Data collection and follow-up

The patients will be followed-up at 45 days, 3 and 6 months, and 1, 2 and 3 years after randomization. The follow-up protocol includes a physical examination to assess wound events, wound pain, patient temperature and adverse events, and a biological investigation (leukocyte count and C-reactive protein). At 6 months and 3 years, a CT scan and a bacteriological investigation are mandatory. These investigations may be performed at any other consultation according to clinical requirements. The quality of life questionnaires EuroQol 5D, SF-12 and CCS will be completed by the patient at each stage of the 3 years of follow-up. The necessity for follow-up beyond the scheduled formal consultations will depend upon the surgeon’s clinical judgment. All events and additional costs will be listed.

### Statistical evaluation and sample size

The hypothesis of this phase III study is that the use of a biological mesh in infected incisional hernias will reduce wound morbidity within 6 months.

The sample size was calculated using the difference in the primary end point of morbidity, which is expected to be 60% in arm B [11-13] and 30% in arm A.

To demonstrate this difference of 30%, using α = 0.05 and β = 0.20, and according to the chi-squared test, the sample size required in each group is 49. This is based on a two-sided significance level (alpha) of 0.05 and a power of 80%. Since we estimate that very few patients will be lost to follow-up because of the short time-scale for the primary end point (6 months), we consequently plan to include 100 patients in this study.

The statistical evaluation will be based for the primary end point on the intention-to-treat analysis set, using a chi-squared test or the Fisher’s exact test. Statistical analysis will be performed with the SAS software version 9.2.

This study is planned to last for 5 years, with a 2-year inclusion period and a 3-year follow-up period; we expect that two patients will be included per year for each participating center. The results for the primary end point will be available 6 months after the end of the inclusion period (2 years).

### Medico-economic analysis

A cost-effectiveness analysis will be performed alongside the clinical evaluation. The direct medical costs (hospital and ambulatory costs) and non-medical costs (transportation costs) will be collected prospectively over the 3-year follow-up period. The patients’ quality of life will be assessed using two generic questionnaires (SF-12 and EuroQol 5D) and the Carolinas comfort scale, a specific, validated questionnaire for patients undergoing hernia surgery. An intermediate analysis will be conducted at 6 months. A Markov model will be constructed using 3 years’ worth of follow-up data to assess the cost-effectiveness of biologic meshes versus standard wound care taking into account all intercurrent events (hernia, infections, encapsulation and so on) that affect both costs and patients’ quality of life.

### Participating centers

To prevent institution bias, the centers participating in this trial are experienced in wound surgery, especially incisional hernias with an active infection. The surgical procedure for biological mesh placement has been sent to each participating center in order to standardize the surgical technique.

In this study, 41 centers will participate: Lille (4 centers), Bordeaux (3 centers), Lyon (4 centers), Montpellier, Nantes, Caen, Rouen, Dijon, Grenoble, Strasbourg, Toulouse, Tours, Clermont-Ferrand, Marseille (2 centers), Angers, Nimes, Besancon, Reims, Rennes, Limoges, Nancy, Amiens, Paris (Saint-Antoine Hospital, Lariboisière Hospital, La Pitié-Salpétrière Hospital, Cochin Hospital, Georges Pompidou European Hospital, Tenon Hospital, Beaujon Hospital Clichy, Kremlin Bicetre, Henri Mondor Hospital Créteil, Ambroise Paré Boulogne-Billancourt).

### Funding, ethics and safety

This research program is funded by the French Ministry of Health through Soutien aux Techniques Innovantes et Coûteuses (STIC) 2011. This study protocol was approved by the national ethics board (the Committee for the Protection of People) on 12 February 2012 under the registration number 2012-A00088-35 and received the authorization of the Agence Française de Sécurité Sanitaire des Produits de Santé on 30 March 2012 under the registration number 2011-A00059-34. The study is registered on the Clinicaltrials.gov website under the identifier NCT01594450.

The study complies with the Declaration of Helsinki and the principles of good clinical practice guidelines. Informed consent is obtained from each patient in written form prior to randomization. The patient is informed about the nature, duration and possible consequences of the trial, by a surgeon familiar with the study, orally and with the help of an information document.

Patient safety and all potential threats to the patients are monitored at each consultation by an independent data safety monitoring board (DSMB), or additionally at the discretion of the DSMB or sponsor; the DSMB will also confidentially evaluate the primary end-point data. Qualified personnel at each sponsor site also meet every 3 months to review the safety data, including adverse events and serious adverse events. Any information deemed to potentially affect the safety of the trial will be brought to the attention of the DSMB.

### Comments

Despite considerable improvements in incisional hernia treatment, infections associated with incisional hernias remain a difficult problem. The use of innovative materials, such as biological meshes, is thought to be the most suitable treatment, due to their specific composition. The non-human collagen matrix can support tissue regeneration through neovascularization and fibroblastic cell repopulation leading to endogenous collagen formation. The exogenous collagen is then resorbed by collagenases. These biological meshes have two main properties: resistance to infection and mechanical resistance, which theoretically facilitate treatment of the infected incisional hernia [8]. The use of biological meshes has expanded rapidly in recent years, despite a lack of substantial evidence and their high cost. No comparative trial is available at the present time, specifically for infected incisional hernias (grade 4 on the VHWG grading system). The most significant study was presented as a poster at the American College of Surgeons Clinical Congress in 2010, which included 80 patients in infected and contaminated fields; however, this was an observational, non-comparative study [14]. A randomized, controlled trial, sponsored by industry, has been designed, comparing the biological mesh Tutomesh to fascia closure without mesh reinforcement in potentially contaminated fields (grade 3 on the VHWG grading system), but the results are still unknown. It is therefore important to evaluate biological meshes, using both medical and economic parameters, for patients most suitable for their use and for whom the cost-benefit balance should be optimal, that is, patients with an incisional ventral hernia with an active infection.

Despite a short-term follow-up (6 months) for the primary end point, the study will collect 3 years of follow-up, which is the longest follow-up available in studies of biological meshes. The choice of a primary end point at 6 months’ follow-up has been chosen for ethical considerations to avoid prolonged treatment without a biologic mesh in patients whose wounds fail to heal.

To conclude, in the SIMBIOSE study, we aim to test the hypothesis that the use of a biological mesh will reduce abdominal morbidity, compared to standard wound care without a biological mesh, in a large, multicenter, phase III, prospective, randomized, controlled, single-blinded trial. Moreover, this trial will assess patients’ quality of life and the medico-economic effects of the different treatment strategies.

## Trial status

At the time of publication the trial is open to recruitment with 41 patients enrolled: the first patient was included in May 2012.

## Abbreviations

CCS: Carolinas comfort scale; DSMB: Data safety monitoring board; CT: Computed tomography; FRENCH: Fédération de recherche en chirurgie (French federation of surgical research); IVH: Incisional ventral hernia; VHWG: Ventral hernia working group.

## Competing interests

The authors declare that they have no competing interests.

## Authors’ contributions

CM, NB, FD, BD, AD, MG, EB, WR and GP designed the trial and developed the protocol; CM, NB, MG, EB and WR drafted the manuscript; AD designed and drafted the statistical plan; BD designed and drafted the economic analysis; CM, NB, FD, BD, AD, MG, EB, WR and GP all revised the manuscript critically. All authors have given their final approval of the version to be published.

## References

[B1] Kingsnorth A LeBlanc K Hernias: inguinal and incisional Lancet 2003 362 1561 71 10.1016/S0140-6736(03)14746-0 14615114

[B2] Luijendijk RW Hop WC van den Tol MP A comparison of suture repair with mesh repair for incisional hernia N Engl J Med 2000 343 392 8 10.1056/NEJM200008103430603 10933738

[B3] Burger JW Luijendijk RW Hop WC Halm JA Verdaasdonk EG Jeekel J Long-term follow-up of a randomized controlled trial of suture versus mesh repair of incisional hernia Ann Surg 2004 240 578 83 15383785 10.1097/01.sla.0000141193.08524.e7 PMC1356459

[B4] den Hartog D Dur AH Tuinebreijer WE Kreis RW Open surgical procedures for incisional hernias Cochrane Database Syst Rev 2008 3 CD006438 10.1002/14651858.CD006438.pub2 PMC8924951 18646155

[B5] Breuing K Butler CE Ferzoco S Incisional ventral hernias: review of the literature and recommendations regarding the grading and technique of repair Surgery 2010 148 544 58 10.1016/j.surg.2010.01.008 20304452

[B6] Choi JJ Palaniappa NC Dallas KB Rudich TB Colon MJ Divino CM Use of mesh during ventral hernia repair in clean-contaminated and contaminated cases: outcomes of 33,832 cases Ann Surg 2012 255 176 80 10.1097/SLA.0b013e31822518e6 21677561

[B7] Cohn SM Giannotti G Ong AW Prospective randomized trial of two wound management strategies for dirty abdominal wounds Ann Surg 2001 233 409 13 10.1097/00000658-200103000-00016 11224630 PMC1421258

[B8] Shankaran V Weber DJ Reed RL 2nd Luchette FA A review of available prosthetics for ventral hernia repair Ann Surg 2011 253 16 26 10.1097/SLA.0b013e3181f9b6e6 21135699

[B9] Blatnik J Jin J Rosen M Abdominal hernia repair with bridging acellular dermal matrix – an expensive hernia sac Am J Surg 2008 196 47 50 10.1016/j.amjsurg.2007.06.035 18466872

[B10] Schuster R Singh J Safadi BY Wren SM The use of acellular dermal matrix for contaminated abdominal wall defects: wound status predicts success Am J Surg 2006 192 594 7 10.1016/j.amjsurg.2006.08.017 17071190

[B11] van’t Riet M de Vos van Steenwijk PJ Bonjer HJ Steyerberg EW Jeekel J Mesh repair for postoperative wound dehiscence in the presence of infection: is absorbable mesh safer than non-absorbable mesh? Hernia 2007 11 409 13 10.1007/s10029-007-0240-5 17551808

[B12] Agag RL Granick MS Omidi M Catrambone J Benevenia J Neurosurgical reconstruction with acellular cadaveric dermal matrix Ann Plast Surg 2004 52 571 7 10.1097/01.sap.0000122651.12811.3d 15166985

[B13] Halm JA de Wall LL Steyerberg EW Jeekel J Lange JF Intraperitoneal polypropylene mesh hernia repair complicates subsequent abdominal surgery World J Surg 2007 31 423 9 10.1007/s00268-006-0317-9 17180562

[B14] Awad S Baumann D Bellows C Prospective multicenter clinical study of single-stage repair of infected or contaminated abdominal incisional hernias using Strattice reconstructive tissue matrix Eur Hernia Soc 2010 http://www.kci-medical.de/cs/Satellite?blobcol=urldata&blobheadername1=Content-type&blobheadername2=Content-disposition&blobheadername3=MDT-Type&blobheadervalue1=application%2Fpdf&blobheadervalue2=inline%3B+filename%3D878%252F901%252FLifeCell_ACS%2B%2528RICH%2529%2Bposter_HandOut_EU_high%2Bres%252C0.pdf&blobheadervalue3=abinary%3B+charset%3DUTF-8&blobkey=id&blobtable=MungoBlobs&blobwhere=1226647231141&ssbinary=true

